# Response of Fungal Diversity, Community Composition, and Functions to Nutrients Management in Red Soil

**DOI:** 10.3390/jof7070554

**Published:** 2021-07-12

**Authors:** Muhammad Atif Muneer, Xiaoman Huang, Wei Hou, Yadong Zhang, Yuanyang Cai, Muhammad Zeeshan Munir, Liangquan Wu, Chaoyuan Zheng

**Affiliations:** 1 College of Resources and Environment/International Magnesium Institute, Fujian Agriculture and Forestry University, Fuzhou 350002, China; m_atifmuneer@yahoo.com (M.A.M.); 3180831046@fafu.edu.cn (X.H.); 3190831051@fafu.edu.cn (W.H.); zyd950@163.com (Y.Z.); liangquan01@163.com (L.W.); 2 College of Plant Science, Jilin University, Changchun 130062, China; caiyy19@mails.jlu.edu.cn; 3 College of Biological Science and Technology, Beijing Forestry University, Beijing 100083, China; zeeshanmunir1270@gmail.com

**Keywords:** fungi, diversity, community composition, nutrients management, red soil, soil property, FUNGuild

## Abstract

Soil fungi play a critical role in plant performance and soil nutrient cycling. However, the understanding of soil fungal community composition and functions in response to different nutrients management practices in red soils remains largely unknown. Here, we investigated the responses of soil fungal communities and functions under conventional farmer fertilization practice (FFP) and different nutrient management practices, i.e., optimization of NPK fertilizer (O) with soil conditioner (O + C), with lime and mushroom residue (O + L + M), and with lime and magnesium fertilizer (O + L + Mg). Illumina high-throughput sequencing was used for fungal identification, while the functional groups were inferred with FUNGuild. Nutrient management practices significantly raised the soil pH to 4.79–5.31 compared with FFP (3.69), and soil pH had the most significant effect (0.989 ***) on fungal communities. Predominant phyla, including *Ascomycota*, *Basidiomycota*, and *Mortierellomycota* were identified in all treatments and accounted for 94% of all fungal communities. The alpha diversity indices significantly increased under nutrients management practices compared with FFP. Co-occurrence network analysis revealed the keystone fungal species in the red soil, i.e., *Ascomycota* (54.04%), *Basidiomycota* (7.58%), *Rozellomycota* (4.55%), and *Chytridiomycota* (4.04%). FUNGuild showed that the relative abundance of arbuscular mycorrhizal fungi and ectomycorrhizal fungi was higher, while pathogenic fungi were lower under nutrient management practices compared with FFP. Our findings have important implications for the understanding of improvement of acidic soils that could significantly improve the soil fungal diversity and functioning in acidic soils.

## 1. Introduction

Soil acidification is a serious threat to global terrestrial habitats and one of the major constraints to agricultural productivity [1,2]. Acidic soils comprise approximately 30% of total land globally, accounting for more than 50% of all arable land [3,4]. It is worth noting that anthropogenic practices such as the extensive use of fertilizers lead to severe problems of greenhouse gas emissions [5] and soil acidification [6]. Soil acidification has been one of the major threats for Chinese intensive agriculture owing to excessive use of nitrogen since the 1980s, and it results in changing the soil physicochemical properties with adverse effects on soil microbial communities [7,8]. Therefore, it is a matter of great interest to find innovative technologies to ameliorate soil acidification for agricultural sustainability.

Recently, more emphasis has been given to balanced fertilization because it is an effective agricultural practice that can improve the plant’s nutritional status, as well as change the soil physicochemical properties and microbial communities [9]. There is also global interest in how the nutrients should be managed on farms [10], as well as the development of sustainable soil nutrient management systems, including the reevaluation of existing management practices [11,12]. Various methods of sustainable soil management have been proposed, including the application of organic and inorganic sources that could lead to a healthy soil environment for better crop yield and functioning of soil microbiota. Liming has been recognized as an important agricultural practice that could increase the soil pH by neutralizing the soil acidity, improve the nutrient availability, provide calcium (Ca^2+^) to the soil for plant uptake, and alleviate the elemental toxicity of some nutrients, especially in topsoil [13,14]. Liming efficiency is further enhanced by the addition of agricultural gypsum [15]. Gypsum has long been used to restore sodic soil. However, more recently, it has been widely applied for highly acidic soils. Gypsum addition significantly ameliorates the Ca^2+^ and reduces the Al^3+^ toxicity, while no effect has been reported on soil pH [16]. Mushroom residue is characterized by a low toxic content while having a higher content of organic matter [17]. Nevertheless, mushroom residue has also been reported to improve the soil structure, with a significant effect on improving the soil pH. Similarly, oyster shell powder has a significant effect on improving the soil pH [18]. These improvements in soil profile result in providing a better soil environment for the functioning of soil microbiota through the addition of lime [13,19], gypsum [12,20], mushroom residue [21], and oyster shell powder [18,22]. Magnesium (Mg) fertilizers have also been used to increase the soil pH, and earlier studies have also shown that their addition into the soil has a significant effect on soil physicochemical properties and microbial communities [23,24]. However, the exact mechanism via which these soil amendments influence the soil fungal community and functioning remains unclear.

Fungi, typically known as obligate aerobes [25,26], have the ability to survive in a variety of environments [27]. Soil fungi are most abundant in agricultural ecosystems [28]. They exhibit a wide range of ecologies and play an important role in nutrient transformation in soil [29]. Fungi, rather than bacteria, are thought to be more important in the decomposition of soil organic matter, particularly in acidic environments [30]. It has been widely suggested that higher soil microbial diversity reflects the improved soil quality, implying better substrate use and nutrient supply [31]. However, soil microbial diversity can be affected by poor management practices and environmental changes, posing possible risks to soil quality and productivity [32]. Hence, certain microbial markers such as microbial biodiversity may be seen as early warning signs of soil depletion or amendment [33,34]. Furthermore, a better understanding of microbial functions could lead to more effective management approaches that could have a positive influence on agricultural ecosystem sustainability and productivity. To the best of our knowledge, the impacts of various nutrients management practices on fungal diversity and functioning in red soils have not been thoroughly investigated.

Red soils are mostly found in southern regions of China and account for 22% of the country’s total land area. These soils are recognized for their low pH and low nutrient content with high Al^3+^ toxicity, resulting in poor soil physicochemical properties with antagonistic effects on plant growth and soil microbiota. However, the effects of nutrients management on soil physicochemical properties, soil fungal diversity and abundance, community composition, and functional diversity have not been well investigated in the red soil of pomelo orchards. Therefore, in the current study, different nutrient management practices, including optimization of NPK fertilizer with lime, gypsum, mushroom residue, oyster shell powder, and magnesium, were chosen for application to the acidic soil of a pomelo orchard. We hypothesized that different nutrient management practices could improve the soil pH and change the soil fungal community composition, with an increase in some beneficial fungi, which could be useful for pomelo productivity. To test this hypothesis, the following questions were addressed: (1) What are the main changes in soil properties due to different nutrients management practices? (2) What are the impacts of these nutrient management practices on fungal diversity and community composition? (3) What are the effects of different nutrient management practices on the functioning of fungal communities? Therefore, in this research, fungal community composition and functions were studied using high-throughput sequencing and FUNGuild under different nutrient management practices in the red soil of southern China.

## 2. Materials and Methods

### 2.1. Field Experimental Setup and Sampling

A field experiment was conducted in Pinghe County (24°02′–24°35′ N, 116°54′–117°31′ E), Zhang Zhou city, Fujian province, Southeast China. This region is characterized by red soil type, subtropical monsoon climate with an average annual temperature of 17.5–21.3 °C, and precipitation of 1600–2000 mm [35].

To check the effects of nutrients management on soil physicochemical properties and fungal community, we set up four treatments: (1) farmer fertilization practice with high NPK input (FFP), (2) optimized NPK (O) with different soil conditioners (C), i.e., gypsum, mushroom residue, and oyster shell powder (O + C), (3) optimized NPK with lime and mushroom residue (O + L + M), and (4) optimized NPK with lime and magnesium (O + L + Mg). The fertilizers used in this study included urea (46% N), diammonium phosphate (42% P_2_O_5_), potassium sulfate (51% K_2_O), lime (75% Ca(OH)_2_), and magnesium sulfate monohydrate (27.5% MgO). The fertilizer application rates are shown in Table 1. Eight year old pomelo trees were selected as the plant material and fertilized in December 2018, February 2019, and April 2019 (Table S1). Each treatment had eight replications, and a total of 32 soil samples were collected in June 2019 from the topsoil (0–20 cm) after removal of the topsoil layer of 5 cm to evade the exogenous disturbance. The soil samples were collected from the fertilizer zone (i.e., for nutrient management, 20–80 cm around the tree trunk; for FFP, around 75–125 cm). Each soil sample was divided into two parts; one part was used for the determination of soil physicochemical properties, and other was stored at −80 °C for molecular analysis.

### 2.2. Determination of Soil Physicochemical Properties

The soil physicochemical properties were determined according to the protocol described by Bao (2000) [36]. In short, pH (soil/water 1:2.5) was measured using a SJ24A-type pH meter. NH_4_^+^-N and NO_3_^−^-N were extracted with 2 mol·L^−1^ KCl and analyzed using a flow analyzer. Available phosphorus (AP) was extracted using HCl–NH_4_F and analyzed using a spectrophotometer. Available potassium (AK) was detected using ammonium acetate extraction and flame photometer analysis. Exchangeable calcium (Ex.Ca) and exchangeable magnesium (Ex.Mg) were extracted with 1 mol·L^−1^ NH_4_Ac and determined using inductively coupled plasma optical emission spectroscopy (ICP-OES).

### 2.3. Soil DNA Extraction

Soil DNA was extracted from approximately 0.5 g of soil with a PowerSoil DNA Extraction Kit (QIAGEN Inc., Valencia, CA, USA) following the manufacturer’s instructions. For the determination of DNA quality and concentration, a NanoDrop-2000 spectrophotometer (Nanodrop Technologies, Wilmington, DE, USA) was used. Agarose gel (1%) was used for measuring the DNA concentration and purity. DNA was diluted with sterile water to the final concentration of 1 ng·µL^−1^.

### 2.4. PCR Amplification and Sequencing

The fungal rDNA ITS1 (internal transcribed spacer 1 of rDNA) region was amplified using the high coverage set of primers ITS1F and ITS2 [37]. The PCR mixture consisted of 15 µL of Master Mix (Takara Biotechnology, Dalian, China), 0.2 µmol·L^−1^ of forward and reverse primer, 10 ng of template DNA, and sterilized double-distilled water (dd-H_2_O) to a final of the volume of 30 µL. The PCR (polymerase chain reaction) was carried out according to the following profile: denaturation at 98 °C for 1 min, followed by 30 cycles of 10 s at 98 °C, 30 s at 50 °C, 60 s at 72 °C, and a final extension at 72 °C for 5 min. The amplified PCR products were detected by 2% agarose gel electrophoresis, and an AxyPrep DNA gel extraction kit (Axygen Biosciences, Union City, CA, USA) was used to purify the PCR products. For quantification of PCR products, QuantiFluor^TM^ Fluorometer (Promega Biotech, Beijing, China) was used. Sequencing was performed by Shanghai Majorbio Bio-pharm Technology (Shanghai, China), using the Illumina MiSeq platform (San Diego, CA, USA).

### 2.5. Sequencing Data Processing

The raw sequences were processed using QIIME (Quantitative Insights into Microbial Ecology (QIIME v1.9.1) [38] with default settings and Uprase v7.0.1090 [39]. OTUs (operational taxonomic units) were clustered at 97% sequence similarity. The representative OTUs were selected and annotated using the RDP Classifier (v2.11) as a reference database. All the sequencing data were deposited to the NCBI SRA database with accession number PRJNA730436.

### 2.6. Statistical and Bioinformatics Analyses

All the downstream statistical analyses were assayed by R software (v4.0.3). The α-diversity among different treatments was evaluated using four indices, i.e., observed index, Chao1 index, ACE index, and Fisher index, through a OTU table for determination of fungal community richness and diversity. To check the significant differences for fungal alpha diversity among different treatments, the KW test (Kruskal–Wallis) was employed. The fungal β-diversity and the differences in fungal community between the different treatments were investigated using PCoA (principal coordinate analysis) as a function of Bray–Curtis dissimilarity. Additionally, to check the statistically significant differences among different treatments, PERMANOVA (permutational analysis of variance) was performed. To investigate the relationship between the fungal communities and soil physicochemical properties, including soil pH, NH_4_^+^-N, NO_3_^−^-N, AP, AK, Ex.Ca, and Ex.Mg, a dbRDA (distance-based redundancy analysis) was performed. The significant effects of soil properties on fungal communities were tested by the Mantel test based on Pearson correlation among the soil properties and Bray–Curtis dissimilarity score.

An SEM (structural equation model) was constructed to quantify the contribution of soil properties (pH, NH_4_^+^-N, NO_3_^−^-N, AP, AK, Ex.Ca, and Ex.Mg) on changes in the α-diversity of fungal communities in the red soil of a pomelo orchard. The overall goodness of fit of the SEM was assessed using the normed chi-square test (χ^2^/DF). The values of normed chi-square (χ^2^/DF = 0–2), normed fit index (NFI > 0.90), and root-mean-square error of approximation (RMSEA < 0.05) confirmed the model’s acceptability [40,41]. We used AMOS 24.0 (SPSS, Chicago, IL, USA) for the construction and analysis of SEM.

The co-occurrence network analysis of fungal communities was performed on the basis of the Spearman correlation. The OTUs were chosen on the basis of robust and significant correlations (ρ > 0.6, *p* < 0.05). The robust correlations were identified through pairwise comparisons of taxa abundance, resulting in a complex network of correlations in which each node represented a phylum and each internode (stand) between the nodes showed a substantial correlation. Gephi V0.9.2 was used for co-occurrence network visualization and modularization analysis. Gephi calculated the network’s topological characteristics, such as average path length (the average value of the shortest path between all possible node pairs in the network), clustering coefficient (the propensity of nodes to link with one another), and modularity index (a calculation of how well the network is distributed into different modules) [42]. In the co-occurrence network, keystone species were described as nodes with a high degree and relative abundance [43,44].

For investigating the functions of the fungal community, FUNGuild was used for the identification of functional groups (guilds) in four representative soil samples (FFP, O + L, O + L + M, and O + L + Mg). The fungal functional group (guild) was determined by FUNGuild v1.0 [45]. The high-throughput sequencing datasets from treatments were evaluated using FUNGuild and mainly divided into three trophic modes according to the feeding habits of fungi, i.e., symbiotroph, saprotroph, and pathotroph.

## 3. Results

### 3.1. Changes in Soil Physicochemical Properties

**Figure 1:**
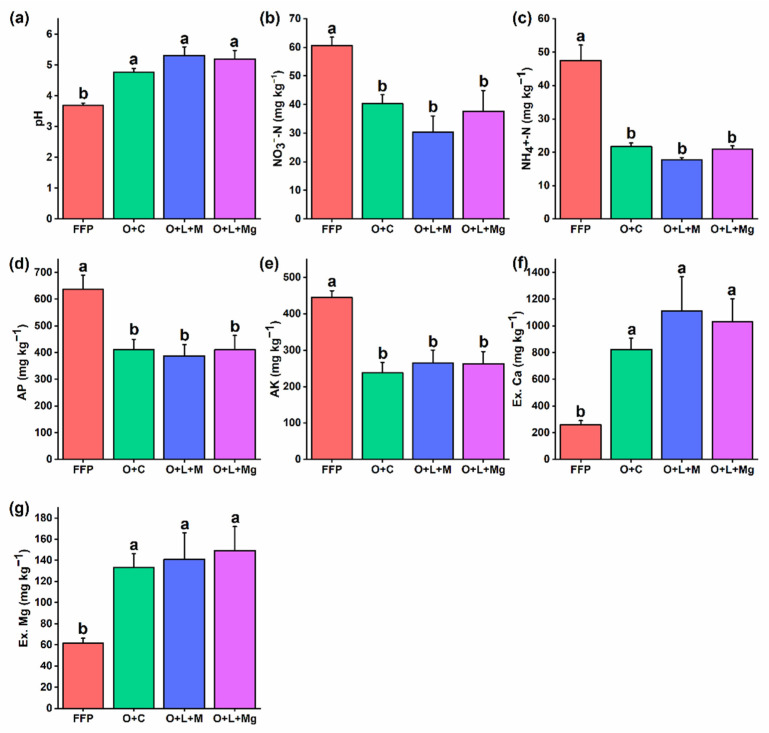
Effect of different management practices on soil properties. The effect of different nutrient management practices was compared with farmer fertilizer practice in terms of soil physicochemical properties: (**a**) soil pH; (**b**) NO_3_^−^-N; (**c**) NH_4_^+^-N; (**d**) available phosphorous; (**e**) available potassium; (**f**) exchangeable calcium; (**g**) exchangeable magnesium. The different lowercase letters indicate significant differences (*p* < 0.05) among different treatments.

Nutrient management practices had a significant effect on improving soil properties. We found that soil pH was significantly higher for O + C (4.77), O + L + M (5.31), and O + L + Mg (5.20) compared with FFP (3.69) (Figure 1a). Furthermore, the soil residual nutrient availability of NO_3_^−^-N, NH_4_^+^-N, AP, and AK under nutrient management practices was significantly lower than FFP because of effective nutrient uptake by plants under high soil pH and resulted in lower residual nutrient availability in the soil (Figure 1b–d). In contrast, the Ex.Ca and Ex.Mg contents were significantly higher under nutrient management practices compared with FFP (Figure 1f,g). Overall, the nutrient management practices were found to have significant positive effects on the soil physicochemical properties.

### 3.2. Effect of Nutrient Management Practices on Fungal Communities

**Figure 2:**
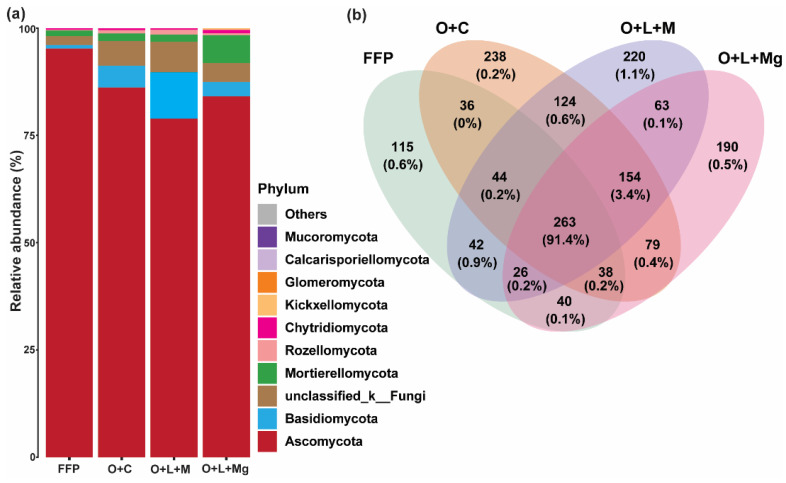
Relative abundance of soil fungal communities. The relative abundance of dominant soil fungal community in different treatments: (**a**) phylum level; (**b**) comparison of different soil fungal community richness. The relative abundance of the top 10 phyla is shown, while less abundant and unclassified phyla are represented by “others”.

Overall, 1672 OTUs were recovered from 32 soil samples and classified on the basis of species (601), genus (407), family (213), order (102), class (42), and phylum (12). The dominant phyla included *Ascomycota*, *Basidiomycota*, *Mortierellomycota*, *Rozellomycota*, and *Chytridiomycota*, accounting for 95% of all fungal communities (Figure 2a). The relative abundance (RA) of *Ascomycota* was higher in FFP compared with other nutrient management practices, while the RA of *Basidiomycota*, *Mortierellomycota*, *Rozellomycota*, *Chytridiomycota*, and unclassified fungi was improved under nutrient management treatments compared with FFP. Nevertheless, these results show that the improved RA of fungal communities at the phylum level was contributed by nutrient management practices. Moreover, the Venn diagram showed that 263 OTUs were common among all treatments, which accounted for 91.4% of total reads (Figure 2b). This suggests the higher similarity of fungal communities among all treatments. However, the unique OTUs were higher under nutrient management practices compared with FFP, i.e., O + C (238) > O + L + M (220) > O + L + Mg (190) > FFP (115) (Figure 2b).

### 3.3. Fungal Richness Increased under Nutrient Management Practices

**Figure 3:**
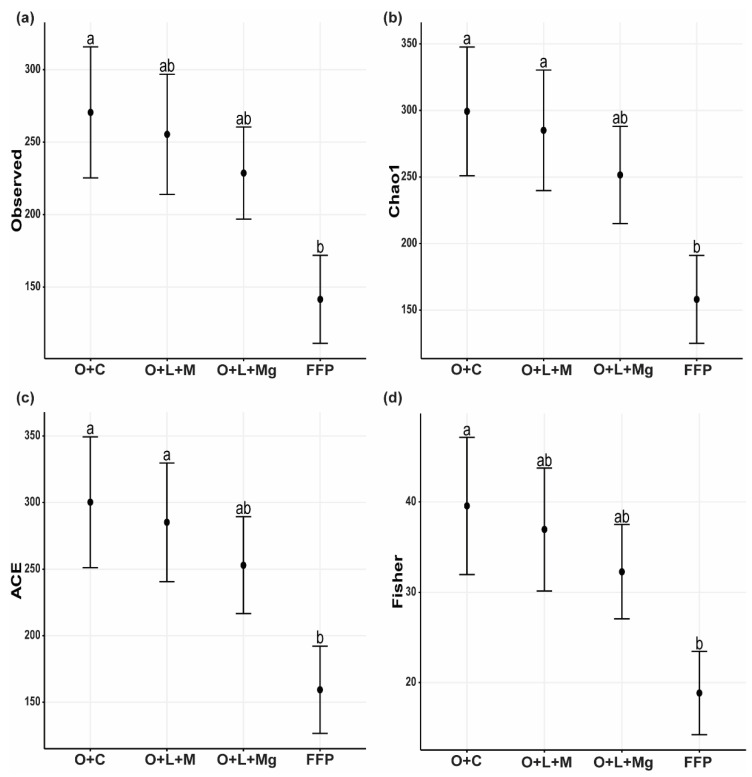
The alpha diversity indices of soil fungal communities. The different α-diversity indices calculated among different treatments: (**a**) observed; (**b**) Chao1; (**c**) ACE; (**d**) Fisher. The different lowercase letters indicate significant differences (*p* < 0.05) among different treatments.

To check the fungal richness, we used four alpha diversity indices, namely, the observed number of OTUs, Chao1, ACE, and Fisher. The fungal richness was significantly higher under the nutrient management practices compared with FFP, i.e., O + C > O + L + M > O + L + Mg > FFP (Figure 3). This implies that nutrient management practices had a significant effect on increasing the species richness, while FFP had a negative effect on fungal species richness.

### 3.4. Changes in Fungal Communities under Different Nutrient Management Practices

**Figure 4:**
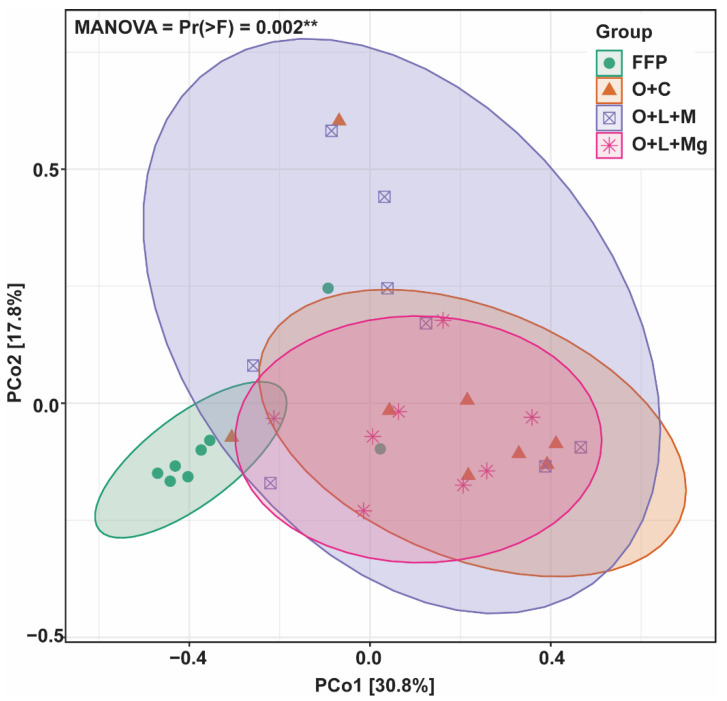
Changes in soil fungal community composition. Principal coordinate analysis (PCoA) showing the differences in fungal communities among different treatments and PERMANOVA (permutational analysis of variance) showing the significant differences among fungal communities of different treatments.

To assess whether nutrient management practices influenced the fungal communities in comparison with FFP, principal coordinate analysis (PCoA) was applied. We found that fungal communities under different nutrient management practices were separated from the FFP, whereby PCo1and PCo2 accounted for 30.8% and 17.8% of the variation (Figure 4). PERMANOVA analysis of all treatments showed significant differences (MANOVA = Pr (>F) = 0.002 **) in fungal communities (Figure 4). Furthermore, we performed the PERMANOVA analysis for each group to evaluate the difference between different treatments and found that fungal communities could be separated (Table 2). Fungal communities under nutrient management practices (i.e., O + C, O + L + M, and O + L + Mg) were significantly different from those under FFP. These results show that nutrient management practices had a significant effect on fungal community composition.

### 3.5. Soil Physicochemical Properties Correlated with Fungal Community

**Figure 5:**
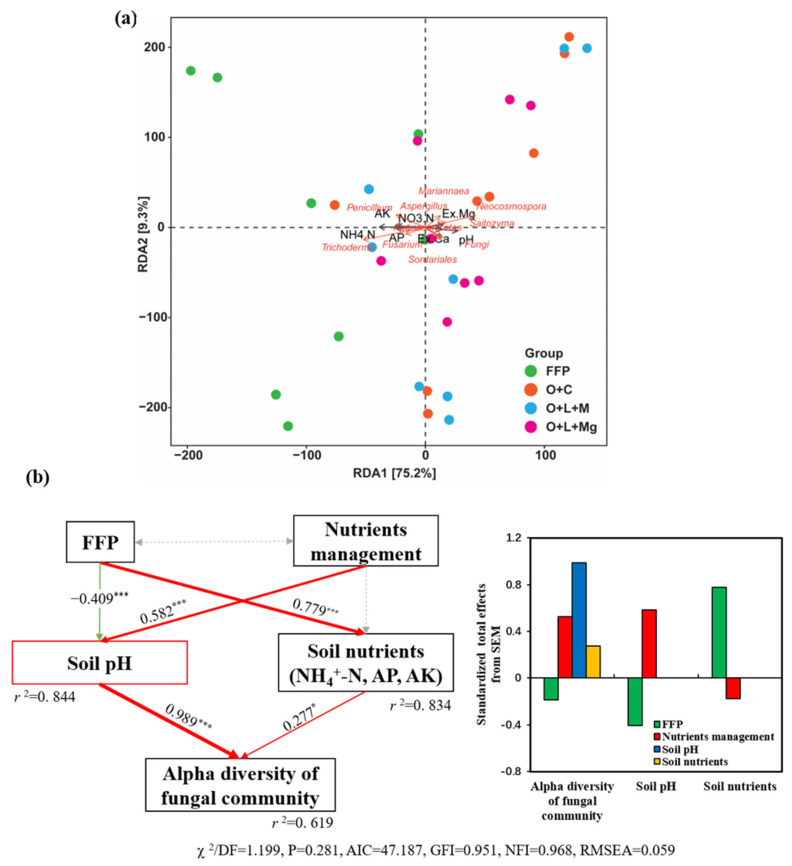
Effect of soil properties on soil fungal communities. The effects of soil properties were evaluated on soil fungal communities: (**a**) distance-based redundancy analysis (RDA); (**b**) structural equation model (SEM). In the SEM, the levels of significance are denoted by * *p* < 0.05, *** *p* < 0.001. The *R^2^* values denote the proportion of the variance explained by each variable. The standardized total effects calculated by the SEM are shown below the SEM. The low value of normed chi-square: (χ ^2^/DF, <2), nonsignificant probability level (*p* > 0.05), low Akaike information criteria (AIC), high normed fit index (NFI > 0.90), and low value of root-mean-square error of approximation (RMSEA < 0.05) listed below the SEMs show that our data matched with the hypothetical models.

A distance-based redundancy analysis (RDA) was performed to assess the effect of soil properties on fungal communities, showing that soil properties, except for NO_3_^−^N, Ex.Ca, and Ex.Mg, were significantly and positively correlated with fungal communities. Specifically, soil pH, NH_4_^+^-N, AP, and AK had significant effects on fungal community composition (Figure 5a, Table 3). A structural equation model (SEM) was constructed to quantify each factor, i.e., soil pH, NH_4_^+^-N, AP, AK, Ex.Ca, and Ex.Mg, concluding that soil pH had the most significant effect (0.989 ***) effect on the alpha diversity of the fungal community for the observed number of species (Figure 5b).

### 3.6. Co-Occurrence Network Analysis

**Figure 6:**
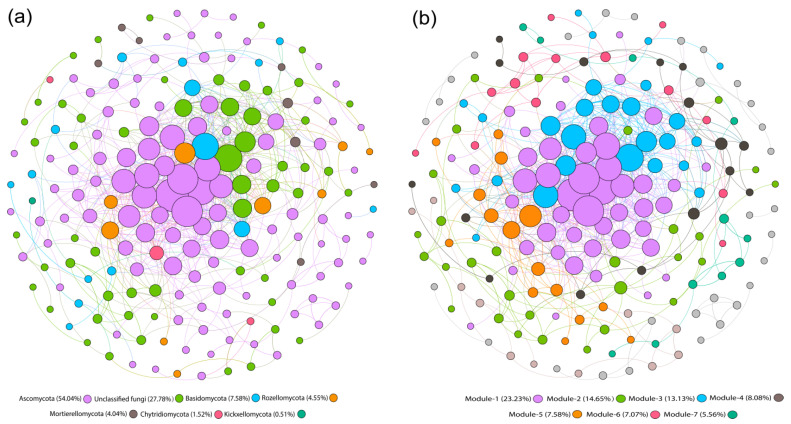
Co-occurrence network of fungal communities in red soil. The co-occurrence network based on correlation analysis for fungal communities: (**a**) the network’s nodes are colored according to their phylum; (**b**) the nodes in the network are colored according to modularity class. The size of each node is proportional to the relative abundance of specific taxa. The connections showing the strong (spearman’s ρ ≥ 0.6) and significant (*p* ≤ 0.05) correlations.

For a better understanding of the taxonomic characteristics of fungal communities in the red soil, a co-occurrence network analysis was performed. The network analysis resulted in 732 edges across 198 nodes and showed a significant correlation between the fungal communities (ρ = 0.61, *p* < 0.05). Furthermore, topological characteristics were measured to evaluate the complex relationship among the nodes [46]. The average path length and diameter were 4.196 and 14 edges, respectively. The modularity index was 0.423, and the modularity index greater than 0.4 shows the presence of modularity in the structure of the co-occurrence network, while the average clustering coefficient was 0.446 [47]. The nodes in the co-occurrence network with a high abundance divided into six phyla (Figure 6a). Among them, *Ascomycota, Basidiomycota, Rozellomycota*, and *Mortierellomycota* accounted for 70% of all nodes and characterized the most dominant fungal communities at the phylum level in the red soil. When the node distribution was modularized, all nodes were primarily divided into seven modules (Figure 6b), and each module comprised a set of OTU nodes.

### 3.7. Functional Prediction Analysis

**Figure 7:**
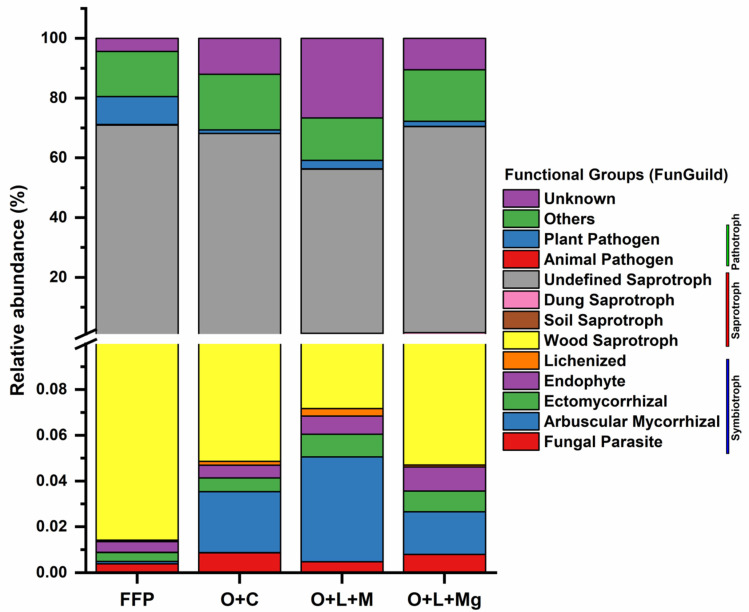
Fungal functional groups inferred by FUNGuild. The variation in the relative abundance of fungal functional groups., i.e., pathotroph, saprotroph, and symbiotroph, was inferred by FUNGuild among different treatments.

FUNGuild analysis was performed for the determination of predicted functions of fungal communities regarding trophic modes in FFP and nutrient management treatments in the red soil of a pomelo orchard, and major functions were classified as symbiotroph, saprotroph, and pathotroph, accounting for an average of 72% of total abundance of predictive functional analysis. We found that, under nutrient management practices, the relative abundance of plant pathogens was decreased compared with FFP. In contrast, under nutrient management practices, the relative abundance of arbuscular mycorrhizal fungi and ectomycorrhizal fungi was higher compared with FFP treatment (Figure 7). This suggests that nutrient management practices had a positive impact on improving the beneficial fungi (e.g., mycorrhizal fungi) and repressing the pathogen fungi (e.g., plant pathogens).

## 4. Discussion

Intensive and inappropriate use of NPK fertilizer leads to serious problems of soil acidification and results in a significant reduction in the soil microbial diversity. However, extensive research is needed to understand the relationship between soil microbiota and soil properties. Soil microbial populations have a significant role in sustaining soil productivity through different biological processes, e.g., residue decomposition and nutrient cycling. Therefore, it is of prime importance to optimize and manage the nutrient input in such a way that it could improve the soil physicochemical properties and crop growth, as well as maximize the soil microbiota diversity.

We found that different nutrient management practices, including O + C, O + L + M, and O + L + Mg, significantly improved the soil pH as compared to FFP. The FFP without lime and gypsum addition significantly decreased the soil pH and exchangeable base cations (Ca^2+^ and Mg^2+^), while the addition of lime and gypsum with reduced input of NPK fertilizer significantly increased the soil pH and exchangeable base cations (Figure 1). It has been reported that the application of inorganic N-fertilizer induces soil acidification [48], whereas lime application reduces soil acidification by effectively decreasing the exchangeable acidic cations [49]. However, we also found that lime application raised the soil pH, and these results are in agreement with the previous findings [50,51]. This could be due to the neutralization of hydrogen ions by hydroxyl groups to form water, while calcium ions may form complex compounds of iron, manganese, and aluminum [49]. Furthermore, despite gypsum’s very slight effect on soil pH, it promotes root growth and development for uptake of nutrients [20]. However, we found that, under FFP, the nutrient content (e.g., NO_3_^−^-N, NH_4_^+^-N, AP, AK) was high. Thus, Al^3+^ toxicity under highly acidic soil environments could be the factor affecting root growth negatively for nutrient uptake [52,53]. In contrast, nutrient management practices increased the soil pH, and this could be useful for root growth, thereby facilitating the plants to take up more nutrients for better plant growth. Hence, we found a significantly lower amount of nutrients in the soil in such environments. It has also been reported that excessive use of NPK fertilizer results in the accumulation of a large amount of nutrients in the soil and leads to deterioration of soil properties [7,54]. Thus, we concluded that nutrient management practices have a significant role in improving soil properties.

The relative abundance of fungal species and alpha diversity was significantly higher under nutrient management practices compared with FFP. Overall, the relative abundance of *Ascomycota* was high under all treatments. *Ascomycota* is known as the most ubiquitous and diverse phylum of eukaryotes, indicating the decomposition of organic substrate [55], and we also found it as a predominant fungal phylum in the red soil of a pomelo orchard. However, the relative abundance of *Basidiomycota* and *Mortierellomycota* was greater under nutrient management practices compared with FFP. *Basidiomycota* comprises some of the most common fungi known for their ability to produce massive fruiting bodies, as well as plant-parasitic fungi that cause wood decay and decomposition of litter [21,56]. This fungal category could be very beneficial to plants owing to their symbiotic association with the roots of the host plant, and they may play a significant role in storing mineral nutrients, metabolites, and water [56]. *Mortierellomycota* was also found in all treatments, but in lower abundance than *Ascomycota* and *Basidiomycota*. The fungal species from this phylum can be found in various environments, including rhizosphere and plant tissues, playing an important role in the carbon cycle and decomposition of organic matter. These fungal species are also recognized as plant growth-promoting fungi [57]. Our findings are consistent with previous research on agricultural soils [58,59] and forest soils [60,61], where *Ascomycota* and *Basidiomycota* were the most prevalent phyla.

The increase in the relative abundance of fungal species under nutrient management practices was contributed by liming, gypsum, mushroom residue, and oyster shell powder. It has been widely accepted that liming positively modulates the soil microbial community composition [62]. This is because lime amendment results in increased soil pH under nutrient management, i.e., 4.8–5.4, and it has been found that fungal species show a wide range of optimum growth in a soil pH of 5–9 [63,64]. The findings of previous investigations are, thus, corroborated by our results, in that lime addition boosts the soil microbial population [65,66], and that lime application significantly increases the relative abundance of soil microbes in red soils [55,67] and positively correlates with soil pH in the range 4.0–8.3 [68]. Thus, liming increases the soil pH and, as a result, decreases the availability of metal ions, e.g., Al^3+^, minimizing the potential toxicity to plant growth and microbe growth [13,19]. Similarly, gypsum application resulted in an improvement in soil properties compared with FFP (Figure 1), consistent with previous findings [12,20]. Nevertheless, gypsum has no direct effect on soil pH but decreases the Cu and Mn availability, as well as increases the P, Ca^2+^, and S-SO_4_^2−^ availability. The ligand exchange reaction of S-SO_4_^2−^ with OH^−^ is associated with Fe and Al oxides, which replaces the OH^−^ and facilitates neutralization of soil acidity, these modifications result in a rise in soil pH [12]. Hence, the addition of gypsum changes the soil properties and consequently leads to a positive effect on soil microbiota. Furthermore, mushroom residue and oyster shell powder also had a positive impact on soil microbial communities, consistent with previous findings [21] that mushroom residue improved the fungal communities. The possible explanation for increasing fungal community under mushroom residue may be related to an improvement of the soil environment, e.g., soil pH [69], thus leading to soil microorganisms having a healthier ecosystem [21,70]. Similarly, we found that oyster shell powder also had a significant effect on improving the soil microbial diversity and community composition, and these results are consistent with previous findings [18,22]. The reason for this may be that oyster shells produce glycosaminoglycan and aspartic proteinases, which can help soil microbes grow faster [18,71], as well as increase the soil pH, thereby providing a better environment for soil microbiota [18]. This study also showed that Mg application increased soil pH and resulted in a positive effect on soil microbes [72].

Moreover, redundancy analysis (RDA) also exhibited that soil pH, NH_4_^+^-N, AP, and AK significantly affected the fungal community structures (Figure 5a, Table 3), and it has been well established that soil pH is the most significant determinant for shaping fungal communities [73,74,75]. The SEM also explained that changes in fungal communities were mainly affected by the soil pH (Figure 5b), and soil pH has been identified as a crucial factor in determining the structure of the soil fungal population [59,68,76]. Soil pH may influence the structure of fungal communities by altering nutrient availability or placing physiological constraints on fungal development [77]. These findings are consistent with previous studies suggesting that fungal communities were significantly changed by the soil pH [78,79].

Furthermore, the interrelationship among the different microbial communities plays a vital role in microbial ecosystem functioning and stability [80]. In this study, the co-occurrence network analysis showed that the fungal communities belonging to phyla *Ascomycota, Basidiomycota, Rozellomycota*, and *Mortierellomycota* were recognized as dominant (Figure 6) according to their highly connected nodes [80,81]. These keystone taxa are critical for preserving the co-occurrence network structure [82], because the loss of keystone species could lead to its disintegration [83]. In addition, the relative abundance of *Chytridiomycota* and *Kickxellomycota* (0.50–1.50%) suggests the significance of rare fungal communities in the red soil. It has been recognized that rare genera of the microbial population are also known as critical components of microbial population assembly and biochemical processes [84]. Although the relative abundance of rare microbial populations is not high, they are a matter of great interest and importance as key nodes in the microbial community [80].

FUNGuild, the functional analysis of fungal communities, revealed that nutrient management practices increased the relative abundance of symbiotroph (e.g., endophyte, arbuscular mycorrhizal, and ectomycorrhizal fungi), whereas they decreased the relative abundance of plant pathotrophic fungi (e.g., plant-pathogen) compared with FFP. Generally, symbiotrophic fungi, in general, are highly helpful for the fitness, nutrition, and sustainability of most crops [85,86,87]. Pathotrophic fungi obtain nutrients by invading host cells. As a result, they are thought to induce disease or have a negative impact on plant efficiency [88]. Our results are consistent with previous findings implying that the optimized nutrient management practices had significant effects on improving the saprotrophic fungi while decreasing the relative abundance of pathotrophic fungi [25,79]. Hence, we found that the nutrient management practices had a positive impact on functional fungal communities.

## 5. Conclusions

**Figure 8:**
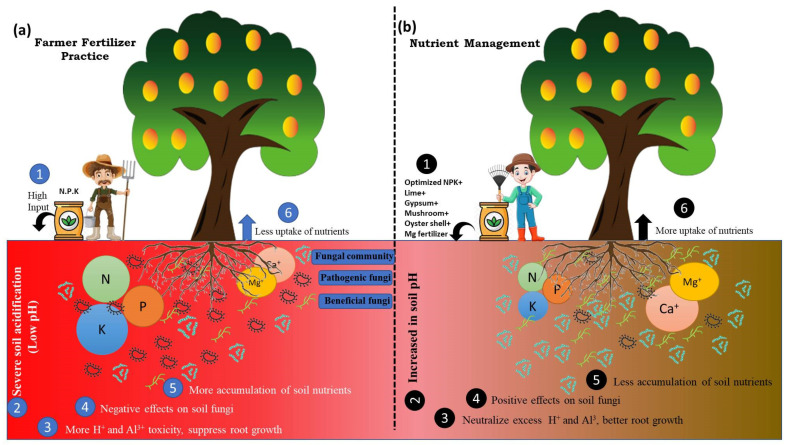
Conceptual framework for explaining how soil properties and fungal communities change under (**a**) farmer fertilizer practice, and (**b**) nutrient management practices.

Taken together, the response of soil fungal communities and their functions to different nutrient management practices was studied by Illumina high-throughput sequencing and FUNGuild. Our results showed that nutrient management practices had a significant effect on soil properties, including soil pH, AP, AK, forms of available nitrogen (NO_3_^−^-N, NH_4_^+^-N), Ex.Ca, and Ex.Mg. Soil fungal richness and diversity significantly increased under nutrient management practices compared with FFP. The fungal community composition was significantly affected by soil physicochemical properties, especially soil pH. The fungal phyla *Ascomycota, Basidiomycota, Rozellomycota*, and *Mortierellomycota* were identified as keystone species in the soil of the pomelo orchard. Furthermore, soil nutrient management practices had a positive impact on increasing the relative abundance of beneficial symbiotrophic fungi, while they decreased the relative abundance of potentially pathogenic fungi. Our findings have important implications for understanding the improvement of acidic soils, and they can provide the basis for future studies on sustainable agricultural measures that could significantly improve the soil fungal diversity, richness, and functioning in acidic soils. In summary, under FFP, the high input of NPK causes severe soil acidification and decreases soil pH. The lower soil pH results in severe soil acidification that negatively affects root growth owing to more H^+^ and Al^3+^ toxicity, leading to plant roots being unable to uptake more nutrients from the soil and, thus, more nutrient accumulation into the soil. Furthermore, the decrease in soil pH also negatively affects the soil fungal communities. In contrast, under nutrient management practices, the higher soil pH has a positive effect on root growth and fungal communities (Figure 8).

## Figures and Tables

**Table 1:** Fertilizer application amount under different treatments.

Treatments	N (kg·ha^−1^)	P_2_O_5_ (kg·ha^−1^)	K_2_O (kg·ha^−1^)	MgO (kg·ha^−1^)	Lime (kg·ha^−1^)	Oyster shell (kg·ha^−1^)	Gypsum (kg·ha^−1^)	Spent Mushroom Residue (kg·ha^−1^)
FFP	867	731	725	0	0	0	0	7700
O + C	128	0	141	0	0	1517	628	2000
O + L + M	128	0	141	0	3108	0	0	2000
O + L + Mg	160	0	160	32	3108	0	0	0

**Table 2:** Significance test of differences among fungal communities under different treatments using permutational analysis of variance based on Bray–Curtis distance.

Groups	Measure	Permutations	*R* ^2^	*p*-Value	Significance
FFP vs. O + C	bray	999	0.260	0.001	***
FFP vs. O + L + M	bray	999	0.207	0.005	**
FFP vs. O + L + Mg	bray	999	0.267	0.001	***
O + C vs. O + L + M	bray	999	0.067	0.410	
O + C vs. O + L + Mg	bray	999	0.046	0.750	
O + L+M vs. O + L + Mg	bray	999	0.098	0.120	

The asterisks represent significant differences (** *p* ≤ 0.01; *** *p* ≤ 0.001).

**Table 3:** Pearson correlation between the Bray–Curtis dissimilarity score and soil characteristics using the Mantel test.

Variable Name	Corr-Method	Corr_Res	*p*_Res	Significance
pH	Pearson	0.192	0.02	*
NO_3_^−^-N	Pearson	0.107	0.088	
NH_4_^+^-N	Pearson	0.251	0.018	*
AP	Pearson	0.17	0.025	*
Ak	Pearson	0.117	0.041	*
Ex.Ca	Pearson	0.06	0.248	
Ex.Mg	Pearson	−0.027	0.546	

The asterisks represent the significance (* *p* ≤ 0.05).

## Data Availability

Not applicable.

## Supplementary Materials

The following are available online at https://www.mdpi.com/article/10.3390/jof7070554/s1, Table S1: Amount of fertilizer applied during different growth periods of pomelo tree.
