# Supplementary material for: Response of Fungal Diversity, Community Composition, and Functions to Nutrients Management in Red Soil
Source: J Fungi (Basel). 2021 Jul 12;7(7):554. doi: 10.3390/jof7070554 (PMC8307627; doi:10.3390/jof7070554)
Supplement: Supplementary file 1 [file jof-07-00554-s001.zip › jof-1245178-supplementary.pdf]

Response of Fungal Diversity, Community Composition, and Functions to Nutrients Management in Red Soil

Muhammad Atif Muneer[1†], Xiaoman Huang[1†], Wei Hou[1], Yadong Zhang[1], Yuanyang Cai[2], Muhammad Zeeshan Munir[3], Liangquan Wu[1], Chaoyuan Zheng[1*]

Table S1. Amount of fertilizer applied during different growth periods of pomelo tree.

		Amount of Fertilizers (kg plant-1)							
	**Treatment**	**Urea**	**Diammonium phosphate**	**Potassium sulfate**	**Lime**	**Mushroom residue**	**Magnesium sulfate monohydrate**	**Oyster shell**	**Gypsum**
**Dec. 2018**	FFP	0.64	0.84	0.69	0	9.91	0	0	0
	O+C	0.13	0	0.13	0	2.57	0	1.95	0.81
	O+L+M	0.13	0	0.13	1.2	2.57	0	0	0
	O+L+Mg	0.17	0	0.15	1.2	0	0.06	0	0
**Feb. 2019**	FFP	0.42	0.56	0.46	0	0	0	0	0
	O+C	0.09	0	0.09	0	0	0	0	0
	O+L+M	0.09	0	0.09	0	0	0	0	0
	O+L+Mg	0.11	0	0.10	0	0	0.04	0	0
**Apr. 2019**	FFP	0.64	0.84	0.69	0	0	0	0	0
	O+C	0.13	0	0.13	0	0	0	0	0
	O+L+M	0.13	0	0.13	1.2	0	0	0	0
	O+L+Mg	0.17	0	0.15	1.2	0	0.06	0	0
